# Elixir Iodo-Bromide Calcium Compound

**Published:** 1875-08

**Authors:** S. P. Crawford

**Affiliations:** Stockton, California


ELIXIR IODO-BROMIDE CALCIUM COMPOUND.—*By S. P. Crawford, A.M., M.D., Stockton, California.*—I fear my lucubrations will become burdensome to the pages of your Journal and wearisome to your readers—so much so that you may be driven to the necessity of striking me from your list of contributors. All I have to say is, "Strike, but hear me," for it has come to the blabbing or bursting point with me.

The above is but one of a class of medicines I see advertised in the medical journals from Dan to Beersheba. Now, a medical journal has been, with me, what a religious newspaper is to our pure and simple-minded church matrons, they look upon their church paper as the next best thing to their Bible, and that its editor is a Paul in learning, faith and purity, and a Peter in piety, and that all the paper contains is sanctioned by this paragon of faith and learning. The paper is looked upon as the expounder of the true faith, and of course could contain nothing in its columns smacking of impiety or falsehood, not even the most vulgar advertisement. Hence it is that the columns of such papers are crowded with advertisements of the vilest quack nostrums, and it is through this class of journals that three-fourths of this class of compounds of stuff and stink find their way to the hearthstones and stomachs of the people.

I look upon the medical journals as being the expounders of the true medical faith, and should contain nothing, inside or outside, that would be calculated to lead the minds of the laity in the profession astray. The great body of the profession have not extensive libraries, nor are they blessed with the means to possess themselves with every new book as it comes steaming from the press, laden with the treasures of the latest discoveries; neither have they time for extensive reading. Their journal is their medical bible, and their main reliance to keep posted on therapeutics and general medical science.

With this exordium I desire to relate my experience with the above article, with some comments added thereto.

I saw the article, whose name appears at the head of this paper, advertised in the pages of all the journals that come under my observation, filling from two to four pages, setting forth its virtues, with such flattering notices from physicians, relating marvelous cures in many cases, etc., that I felt anxious to try it, believing, from the flattering encomiums passed upon it by those who had tried it, that it was certainly the *desideratum* in certain diseases. I made inquiry for it in the shops, but failed to get it. I wrote to the proprietors, asking price and where it could be had, and was informed that it was for sale in all the principal drug stores in the United States. Not long after this I had a case that had run the rounds of the advertising quacks and through the gauntlet of nostrum-vendors, from a pinch of dry "monkey-dung" to the "last chance." The case was such an one as I thought might be met by the above article. Before taking the case I made my patient promise to abandon all nostrums, not to listen to the croaking of old women, nor be allured by the advertising quacks or "traveling doctors," but if any should come about to knock them on the head with a pine knot or set the dogs on them; I made my patient promise implicitly to follow my directions, or I would not take the case.

Having thus fortified myself against all intruders, I gave a little medicine preparatory to the *coup de main* with the Elixir Iodo-Bromide Calcium Compound.

Three or four days after this I called and prescribed the aforementioned article, and ordered it to be in readiness at my next visit.

*It* came and *I* came at the appointed time. My first inquiry, after the salutations of the morning, was, "have you got the medicine ordered?" I never shall forget the look of reproach and disappointment of my patient as she answered in the affirmative, pointing to a bottle standing on the shelf. It stood by the side of an empty bottle of Jayne's Expectorant. It was pasted all over, from stem to stern, with yellow paper, giving its ingredients, setting forth its virtues in a host of diseases, directions as to dose, etc., flattering notices from medical journals, and encomiums from numerous physicians, extolling its wonderful virtues.

Along with this pasted cover was a whole lot of loose wrapper, containing pretty much the same things, in a little more extended form. I never saw the vilest compound in the domain of charlatanism more thoroughly fortified with recommendations and flattering notices such as are designed for the gaping public.

My first thought was, Is there not some mistake? I took it down, turned it over and over, looked at it, tasted it, smelt it. No, there is no mistake. Here is the name, occupying the whole length of the bottle, and here are the ingredients. About one-fourth of the contents of the bottle was gone, and I was informed that it was the last bottle in the drug-store, and that some had been sold out of it. Some doctor, no doubt, more fortunate than myself, desiring to test its virtues, had called for it in person, and, not liking too much of a good thing in the way of flattery and recommendation, had taken, a few ounces of it, ashamed to be seen with the original bottle.

"Doctor," said my patient, "I thought you were opposed to patent medicines and all advertised nostrums! You made me promise to have nothing to do with them, and here you are giving the very things you denounced in unmeasured terms!"

I sunk right down into my boots—I wilted—and felt small enough to crawl through a key-hole, and mad enought to bite "pi." "Madam," I said, "this is no patent medicine or advertised nostrum."

"If *it* is not, then *Jayne's* is not, for I can see no difference between it and Jayne's, except that Jayne's is the most neatly labeled. They are both covered with directions as to dose, diseases which they are said to cure, certificates and recommendations."

I used to consider myself pretty good at splitting metaphysical hairs, but here was a medico-therapeutic hair that defied all my skill. I thought I had the advantage in the fact that my hero was eviscerated and the contents of his abdomen were spread out on the label; but these contents were all Greek to my patient.

As a *finale* my patient said: "Doctor, if I am to take this medicine, I can dispense with your services; for all the directions are given here, and I see no further necessity for your attendance."

I left, and up to date I have heard nothing more from my patient.

Now, Messrs. Editors, you see the reason why I want to be heard, and why it is that my wrath has arisen to the point that I must "blab or bust." If ever a man was justifiable in hiring some one to "cuss," I ought to be allowed to hire a score eloquent in the dialect, and then the subject would not be half ventilated. "Why?—because you have lost a patient, and thereby a fee?" Not so; "he that steals my purse, steals trash," but he that steals my good name, let him be *anathema maranatha.* This dabbling with the formidable article at the head of this paper, it is true, did rob me of a patient and a fee; but it did more—it robbed me of a good name. Instead of proving myself an honest physician, it placed me, in the estimation of some, at least, as the most consummate ass in charlatanism. To set my face, like flint, against every appearance of evil in medicine, honestly trying to relieve the sick, has been the business of nearly thirty years of my life. While thus engaged, to find myself plunged, head and ears, into the cesspool of charlatanism, and doused about until the breath of honesty becomes extinct, is a hard road to travel. It is time for all such to "cuss out and quit."

Of the merits or virtues of the article in question I know nothing; therefore have nothing to say about it, *pro* or *con*.

This is what I want to say: If there are any virtues in that medicine, and the proprietors think they have discovered a happy combination of ingredients of value to the profession, and design it for their use, let them prepare it as other medicines are prepared, with simple name upon the label and the name of the house that prepares it. The answer to this is, that it is prepared in bulk bottles of 5 lbs. each for physicians. This is killing two birds with one stone. The proprietor not only prepares it for physicians, but prescribes it in diseases, not in the way physicians usually prescribe medicines, but as the quacks do, by sending it broadcast over the land, fortified by the most flattering encomiums, and specific directions for public use, by any one who may be captivated by its recommendations, as all quack nostrums are.

If this is not quackery, in the name of heaven and all honesty what is it?

The defense, perhaps, for this mode of procedure is, that all these notices, encomiums, specific directions and laudations are designed only for the eyes of the profession. While this may be true, it is plain to be seen that behind all this there lies the desire to thrust it on the public for general and indiscriminate use, as all proprietary medicines are, with the double advantage of being backed up by the profession. If this is legitimate medicine, deliver me from all such! When the business of the Doctor is reduced to the prescribing of this one's compound and that one's elixir, and then, as soon as prescribed, to have the proprietor step in, flourishing his gilded and magic bottle in your face, take charge of the case, kick you, *a posteriori*, and send you home, "I want none in mine." Give me the good old days when doctors kept their own shops and compounded their own medicines.

Having finished my talk, I shall now take a bath, "potenized with the Extract of Moonshine," cuss out and quit.—*Nashville Journal of Medicine and Surgery.*

